# A Novel Approach for the Manufacturing of Gelatin-Methacryloyl

**DOI:** 10.3390/polym14245424

**Published:** 2022-12-11

**Authors:** David Grijalva Garces, Carsten Philipp Radtke, Jürgen Hubbuch

**Affiliations:** 1 Institute of Functional Interfaces, Karlsruhe Institute of Technology, 76344 Eggenstein-Leopoldshafen, Germany; 2 Institute of Process Engineering in Life Sciences Section IV: Biomolecular Separation Engineering, Karlsruhe Institute of Technology, 76131 Karlsruhe, Germany

**Keywords:** biomaterials, cell culture, fibroblasts, gelatin, GelMA, hydrogel, tissue engineering

## Abstract

Gelatin and its derivatives contain cell adhesion moieties as well as sites that enable proteolytic degradation, thus allowing cellular proliferation and migration. The processing of gelatin to its derivatives and/or gelatin-containing products is challenged by its gelation below 30 ${}^{\circ }C$. In this study, a novel strategy was developed for the dissolution and subsequent modification of gelatin to its derivative gelatin-methacryloyl (GelMA). This approach was based on the presence of urea in the buffer media, which enabled the processing at room temperature, i.e., lower than the sol–gel transition point of the gelatin solutions. The degree of functionalization was controlled by the ratio of reactant volume to the gelatin concentration. Hydrogels with tailored mechanical properties were produced by variations of the GelMA concentration and its degree of functionalization. Moreover, the biocompatibility of hydrogels was assessed and compared to hydrogels formulated with GelMA produced by the conventional method. NIH 3T3 fibroblasts were seeded onto hydrogels and the viability showed no difference from the control after a three-day incubation period.

## 1. Introduction

Hydrogels are widely used as scaffolds for tissue engineering (TE) as the polymeric network resembles the extracellular matrix (ECM) within native tissue. For this purpose, hydrophilic polymers are covalently or physically crosslinked in order to create a water-swollen network [1,2]. The building blocks for hydrogel formulation can be purified from native tissue or produced synthetically. Naturally derived polymers show higher biocompatibility and can promote cellular adhesion and proliferation, whereas synthetic polymers have the advantage of low batch-to-batch variation compared to polymers extracted from natural sources [3,4]. The composition of the network influences cellular behavior not only by biochemical cues but also by the resulting physical structure and mechanical properties of the surrounding matrix [5,6].

Gelatin is derived from collagen, the most abundant protein in the native ECM, by acidic or alkaline treatment. The use of gelatin as a cell culture carrier is limited by the sol–gel transition of gelatin solutions at physiological temperatures [7]. In order to increase the stability of gelatin hydrogels at higher temperatures, the protein backbone can be chemically crosslinked. One approach for this purpose is the functionalization of gelatin with methacrylamide and methacrylate residues [8]. The resulting product, gelatin methacryloyl, also known as GelMA, retains sites for cell adhesion as well as for enzymatic degradation that are present in collagen and gelatin [9]. The use of photo-crosslinkable GelMA has gained popularity in the fields of TE and biofabrication [9]. The versatile GelMA has been used in cancer research [10], drug encapsulation and delivery [11], and in bioprinting. Therefore, bioinks containing solely GelMA are used [12], as well as bioink blends with other proteins like collagen [13] or in combination with polysaccharides such as alginate and gellan gum [14,15].

To meet the increasing demand for biomaterials required for TE applications, the manufacturing of hydrogel components requires a thorough understanding of the process parameters to reach a certain quality attribute [16]. In the case of GelMA, an aspect of particular interest is the degree of functionalization (DoF) of the biopolymer as the functional residues are relevant during photo-crosslinking. Since GelMA was first introduced by Van den Bulcke [8] over two decades ago, lab-scale studies have shown increasing reproducibility and efficiency during GelMA manufacturing, where aqueous gelatin in aqueous solution reacts with methacrylic anhydride (MAA). Lee [17] and Shirahama [18] studied the effect of the pH of the protein solution on the produced GelMA. As reaction by-products are acidic, the pH of the solution decreases, thus inhibiting the progress of the reaction. By introducing a carbonate-bicarbonate (CB) buffer at the isoelectric point (IEP) of type A gelatin, these methods showed a reduction of MAA excess over free amino groups from 30- to 2.2-fold. The study by Shirahama [18] also observed the effect of reaction temperature, which proved to have no influence on the DoF. This study was limited to the range of 35–50 ${}^{\circ }C$ as a lower temperature would allow the solution to form a physical gel, thus leading to inefficient mixing and distribution of reactants. To the best of our knowledge, no studies have reported the possibility of GelMA production at temperatures lower than 35 ${}^{\circ }C$.

In this work, we propose a GelMA manufacturing process including a chaotropic salt, i.e., urea, as a buffer component at pH 9 that enables the processing of gelatin at room temperature. GelMA with various degrees of functionalization was produced and used as the basis for hydrogel formulation. Furthermore, the effect of polymer concentration and DoF on the elasticity and swelling behavior of hydrogels was characterized. Additionally, the suitability of GelMA hydrogels for cell culture was assessed by the cultivation of NIH 3T3 fibroblasts. Therefore, an automated image processing workflow was developed for the identification of single cells and the determination of cell viability. Fibroblasts were incubated on GelMA hydrogels produced by the presented method for a three-day period. GelMA produced by the conventional method was used as a control.

## 2. Materials and Methods

### 2.1. GelMA Manufacturing and Characterization

#### 2.1.1. Synthesis and Purification

**Figure 1:**
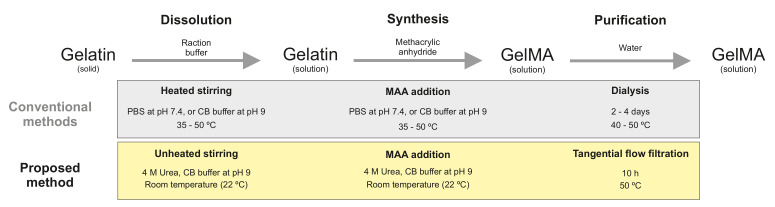
Synthesis and purification of gelatin methacryloyl (GelMA). Conventional methods require the heating of the phosphate-buffered saline (PBS) or the carbonate-bicarbonate (CB) buffer for the dissolution of gelatin. Similarly, the synthesis of GelMA requires heated stirring of the biopolymer solution in order to ensure homogeneous mixing and distribution of the added methacrylic anhydride (MAA). Reaction by-products as well as buffer salts are removed using a dialysis membrane at temperatures above the gelation temperature of GelMA [8,17,18]. The presented method allows for the dissolution of gelatin and the synthesis of GelMA at room temperature under stirring as the urea-containing CB buffer inhibits the formation of a physical gel. Subsequently, GelMA is purified in a tangential flow filtration unit at 50 ${}^{\circ }C$, thereby reducing the processing time to hours.

For the dissolution of gelatin and subsequent synthesis of GelMA, a 0.25 M carbonate-bicarbonate (CB) buffer was prepared according to the method of Lee [17] and Shirahama [18]. Buffer components were purchased from Merck (Darmstadt, Germany). CB buffer was composed of 0.023 M sodium carbonate and 0.227 M sodium bicarbonate, pH 9 was adjusted with 1 M sodium hydroxide (NaOH) or 1 M hydrochloridic acid (HCl). This buffer system was used for the dissolution of gelatin (Type A, 300 bloom strength, Sigma-Aldrich, St. Louis, MI, USA) to a concentration of 10% (*w*/*v*) at 40 ${}^{\circ }C$ under stirring. Subsequently, the reaction was performed at 40 ${}^{\circ }C$ by the addition of methacrylic anhydride (MAA, 94, Sigma-Aldrich) dropwise with a syringe pump (Nemesys, CETONI, Korbussen, Germany) over a period of 100 min. The MAA-to-gelatin ratio was 100 μL g^−1^. The reaction was carried out for additional 20 min. The process was terminated by 2-fold dilution with ultrapure water and pH adjustment to pH 7.4. The reaction mixture was purified with 3.5 kDa molecular weight cutoff dialysis tubing (Thermo Fisher Scientific, Waltham, MA, USA) in an ultrapure water reservoir for 4 days at 40 ${}^{\circ }C$. The conductivity of the water in the reservoir was measured with a conductivity meter (CDM230, Radiometer Analytical SAS, Villeurbanne, France). By the end of the dialysis, the conductivity of the reservoir equaled the conductivity of fresh ultrapure water with a value of 5 μS cm^−1^. This approach for the production of GelMA is referred to as the conventional approach throughout this manuscript. A second buffer system comprised of 0.25 M CB buffer and 4 M urea (Merck) was prepared. The pH of the solution was adjusted to pH 9. In the urea-containing CB buffer, gelatin was dissolved to 10% (*w*/*v*) at room temperature (RT) under stirring, no external heating sources were used. Subsequently, the reaction started by adding MAA dropwise to the appropriate amount. A summary of the produced samples within this study is provided in Table 1. The reaction mixture was then purified against ultrapure water in a tangential flow filtration (TFF) unit (Tandem 1082, Sartorius, Göttingen, Germany) equipped with a 2 kDa molecular weight cutoff membrane (Vivaflow^®^ 200, Hydrosart^®^, Sartorius). The TFF process took place at 50 ${}^{\circ }C$. During purification, the retentate pressure was set to 0.2 MPa, as recommended by the manufacturer as the maximal operating pressure. The purification was stopped after 10 diafiltration volumes. The conductivity of the retentate was equal to the conductivity of ultrapure water by the end of the purification. After each run, the TFF membrane was cleaned with ultrapure water, a 0.1 M sodium hydroxide solution, and a 15 (*v/v*) ethanol (Merck) solution. The GelMA solutions produced by both methods were frozen at −80 ${}^{\circ }C$ and lyophilized at −55 ${}^{\circ }C$ and 0.66 Pa for 4 days. Solid GelMA was stored at RT until further use. The methodology used for the synthesis and purification of GelMA in this study is schematically presented in Figure 1.

#### 2.1.2. DoF Determination

The degree of functionalization (DoF) was determined based on the trinitrobenzenesulfonic (TNBS) acid (Sigma-Aldrich) method by Habeeb [19]. A 0.1 M CB Buffer (0.009 M sodium carbonate, 0.091 M sodium bicarbonate) was prepared and used as a reaction buffer containing 0.01% (*w*/*v*) TNBS. Glycine (Sigma-Aldrich) standards, a gelatin reference, and GelMA samples were dissolved in ultrapure water. A volume of 250 μL of each sample was mixed with an equal volume of the TNBS reagent solution and incubated for 2 h at 40 ${}^{\circ }C$. The reaction was stopped by addition of 250 μL of a 10% (*w*/*v*) sodium dodecyl sulfate (Sigma-Aldrich) solution and 125 μL of a 1 M HCl solution. The absorbance of each sample was measured at 335 nm using a microplate reader (infiniteM200, Tecan Group, Männedorf, Switzerland). The gelatin and GelMA samples were prepared at 0.1, 0.3, 0.5 and 0.8 mg mL^−1^ The concentration of primary amino groups in the samples was determined in comparison to a glycine standard curve, which was measured at 3, 5, 8, 10, and 20 μg mL^−1^ and normalized to the respective protein concentration. The DoF was estimated as the difference between the number of free amines present in gelatin ($c_{NH_{2},\mathrm{gelatin}}$), i.e., before the functionalization, and the amount in the produced GelMA ($c_{NH_{2},\mathrm{GelMA}}$), i.e., after the reaction, divided by the number of free amines in the raw gelatin, as shown in Equation (1).

(1) $$\mathrm{Degree}\mathrm{of}\mathrm{Functionalization}/\text{-}=\frac{c_{NH_{2},\mathrm{gelatin}}-c_{NH_{2},\mathrm{GelMA}}}{c_{NH_{2},\mathrm{gelatin}}}$$

The experimental setup for the characterization of the synthesized GelMA samples consisted of three experimental runs at each MAA-to-gelatin ratio. The absorbance measurements were performed for each independently synthesized batch.

### 2.2. Hydrogel Characterization

#### 2.2.1. Preparation

Prior to the addition of GelMA, the photo-initiator lithium phenyl-2,4,6-trimethylbenzoylphosphinate (LAP, Sigma-Aldrich) was dissolved to the final concentration of 0.5% (*w*/*v*) in Dulbecco’s phosphate buffered saline (DPBS, without calcium and magnesium, 1x, pH 7.4, Thermo Fisher Scientific). GelMA synthesized at different MAA/gelatin ratios was used for hydrogel preparation by dissolving the lyophilized material to 5, 10 and 15% (*w*/*v*). The resulting hydrogel precursor solution was incubated at 37 ${}^{\circ }C$ until complete dissolution of GelMA. A volume of 235 μL GelMA solution was transferred to cylindrical polytetrafluoroethylene (PTFE) molds (diameter 10 mm, height 3 mm). The samples were polymerized by exposure to a UV lamp (365 nm, OSRAM, Munich, Germany) with an irradiation dose of 2850 mJ cm^−2^. Polymerized GelMA hydrogels were incubated in DPBS until further analysis.

#### 2.2.2. Physical Characterization

The rheological and swelling behavior of the hydrogels were characterized in this study. The viscoelastic properties of cell-free hydrogels were characterized based on their storage and loss modulus as a function of frequency. The cylindrical hydrogels were placed between the plate-plate geometry (diameter 10 mm) of a rotational rheometer (Physica MCR301, Anton Paar, Graz, Austria). The gap height was adjusted to 2.5 mm. All conditions were tested within the linear viscoelastic (LVE) regime covering the frequency range of 0.5 to 50 rad s^−1^ at a stress amplitude of 0.5 Pa.

The swelling behavior of the crosslinked hydrogels was characterized by the ratio of the weight of the hydrogel in the swollen state ($m_{\mathrm{swollen}}$) to the weight in the dry state ($m_{\mathrm{dry}}$), as shown in Equation (2). The weight in the swollen state was determined after the equilibration of hydrogels in DPBS. In order to weigh the samples in the dry state, GelMA hydrogels were frozen at −80 ${}^{\circ }C$ overnight and lyophilized for 24 h.

(2) $$\mathrm{Swelling}\mathrm{Ratio}/\text{-}=\frac{m_{\mathrm{swollen}}}{m_{\mathrm{dry}}}$$

Data for the physical characterization study shown below were acquired from measurements performed with three experimental runs with a set of three samples (i.e., technical replicates) each. For each experimental run, GelMA hydrogels from independently synthesized batches were prepared.

### 2.3. Biocompatibility Assessment

#### 2.3.1. Cell Culture

Culture media and supplements were purchased from Gibco™ (Thermo Fisher Scientific). NIH 3T3 mouse fibroblasts (CLS Cell Lines Service, Eppelheim, Germany) were cultured in Dulbecco’s Modified Eagle Medium (DMEM, high glucose, GlutaMAX™) supplemented with 10% (*v/v*) FBS (fetal bovine serum), 50 U mL^−1^ penicillin, and 50 μg mL^−1^ streptomycin. Cells were seeded in tissue culture flasks and maintained at 37 ${}^{\circ }C$ in a humidified, 5% ${\mathrm{CO}}_{2}$ atmosphere. Subcultivation proceeded at 70 to 80% confluence.

#### 2.3.2. Cell Seeding on Hydrogel Coated Well Plates

The biocompatibility of GelMA hydrogels manufactured at room temperature was investigated. Hydrogels formulated with GelMA produced by the conventional method were used as a control. For these experiments, hydrogels were prepared with GelMA at a reactant ratio of 100 μL MAA per gram gelatin. Lyophilized GelMA was dissolved in ultrapure water to a 2% (*w*/*v*) solution at 50 ${}^{\circ }C$. Warm GelMA solutions were sterile filtered using 0.2 μm polyethersulfone (PES) filters (diameter 50 mm, Merck) in a laminar flow cabinet. The sterile solutions were frozen at −80 ${}^{\circ }C$ and lyophilized as described in Section 2.1.1. Hydrogel precursor solutions were then prepared at a concentration of 10% (*w*/*v*) as mentioned in Section 2.2.1. The bottoms of 12-well plates (Thermo Fisher) with a surface area of 3.5 cm^2^ were coated with GelMA by transferring a volume of 350 μL to each well and a subsequent crosslinking under UV exposure. Cells were detached from culture flasks with Trypsin/ethyleneaminetetraacetic acid (Gibco), centrifuged, and resuspended in fresh media. The seeding density on the hydrogel-coated plates was set to 80 × 10^3^ cells per well within a total media volume of 2 mL. Cell-laden samples were kept at incubation conditions until further analysis.

#### 2.3.3. Cell Staining and Imaging

The biocompatibility was assessed after one and after three days of incubation. Staining compounds were purchased from Thermo Fisher (Invitrogen, Waltham, MA, USA). The cell-laden samples were washed with DPBS prior to staining. The samples were covered with 2 mL of staining solution comprised of calcein-AM, propidium iodide, and Hoechst 33342 with concentrations 0.1, 1.5 and 1.66 μM, respectively. Incubation followed for 15 min at 37 ${}^{\circ }C$. Imaging was performed immediately after staining using an inverted microscope (Axio Observer.Z1, Carl Zeiss, Oberkochen, Germany).

#### 2.3.4. Cell Counting and Viability

**Figure 2:**
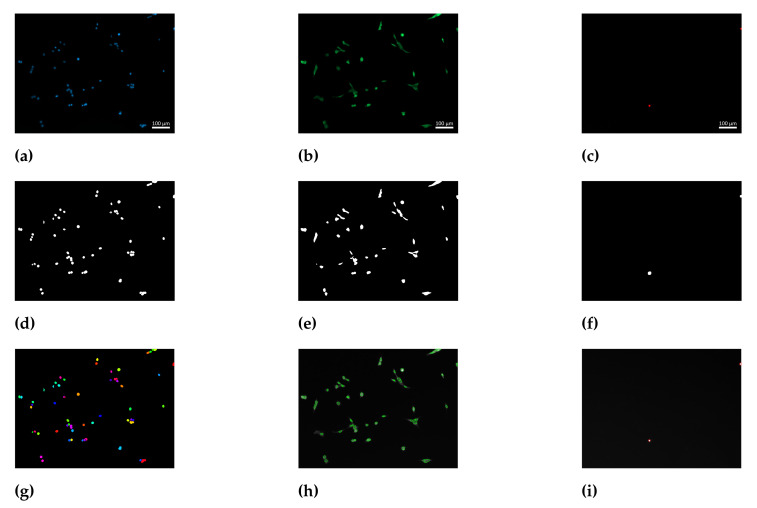
Image processing workflow for cell counting. (**a**–**c**) Single signal images used as inputs for the image processing workflow. Scale bar: 100 µm. (**a**) Nuclear DNA stained using membrane- permeable Hoechst 33342, (**b**) calcein-AM converted to calcein and retained in the cytoplasm, and (**c**) DNA stained using membrane-impermeable propidium iodide. The input images are preprocessed to binary data. (**d**–**f**) Resulting binary images of the regions of interest. (**d**) Nuclear region within all cells, (**e**) cytoplasmic region within viable cells, and (**f**) nuclear region within dead cells. (**g**) Single nuclear regions are identified with a watershed segmentation algorithm. The identified nuclei behind the produced mask from the calcein stain are considered viable, whereas nuclei behind the mask from the propidium iodide stain are considered dead cells. (**h**) Outline of nuclei of cells identified as viable shown in green as a composite with the raw image. (**i**) Outline of nuclei of cells identified as dead shown in red as a composite with the raw image.

In order to obtain an objective determination of the number of cells, an image processing workflow was developed using Matlab^®^ R2021b (TheMathWorks, Natick, MA, USA). The acquired green signal originated from calcein retained within the cellular membrane, whereas the red signal arose from stained DNA of cells with compromised membranes. The blue signal of stained nuclear DNA is present in all cells, both viable and dead, as Hoechst 33342 is membrane permeable. The three signals were used as input for the image processing workflow. The preprocessing of the images consisted of individual binarisation of each signal. The identification of single nuclear regions on the binary image of the blue channel was performed by the watershed segmentation method. Binary images from the green and red signals were used as masks on the blue signal. Nuclei behind the green mask and behind the red mask were considered nuclei of viable and dead cells, respectively. The image processing workflow is shown schematically in Figure 2. Cell viability was calculated according to Equation (3), where the number of viable cells ($N_{\mathrm{viable}}$) was divided by the total number of cells consisting of both viable and dead cells ($N_{\mathrm{dead}}$). The experimental setup consisted of three experimental runs (i.e., biological replicates). For each biological replicate, GelMA precursor solutions from independently synthesized batches were used. Two independent hydrogel samples (i.e., technical replicates) and at least 6 images were recorded of each sample.

(3) $$\mathrm{Cell}\mathrm{viability}/\%=100*\frac{N_{\mathrm{viable}}}{N_{\mathrm{viable}}+N_{\mathrm{dead}}}$$

### 2.4. Data Handling and Statistical Analysis

Data evaluation, image processing, statistical analysis, and data visualization were done with Matlab^®^ R2021b. Results are shown as a mean ± standard deviation. The normal distribution of data sets was verified using the Jarque–Bera test with the α-value set to 0.05. A one-way analysis of variance (ANOVA) was performed in order to find significant differences. A *p*-value below 0.05 was classified as statistically significant.

## 3. Results and Discussion

### 3.1. GelMA Synthesis and Characterization

**Figure 3:**
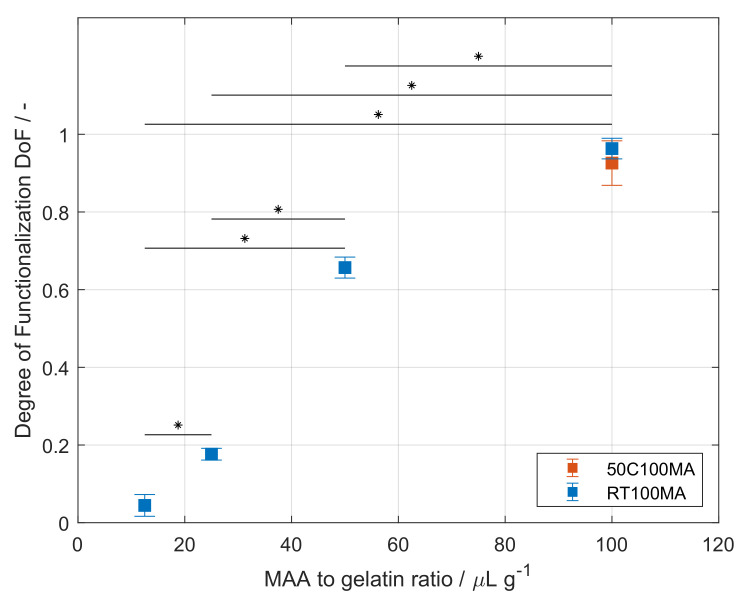
Degree of functionalization (DoF) of produced gelatin methacryloyl (GelMA) batches the DoF was determined by the TNBS method [19]. No difference was found between samples produced a room temperature and samples produced at 50 ${}^{\circ }C$ at 100 µL g^−1^. The DoF increased significantly with increasing the methacrylic anhydride (MAA)-to-gelatin ratio. Asterisks denote a significant difference between samples (p<0.05).

In order to use the biopolymer as a cell culture carrier, gelatin was modified to GelMA. Type A Gelatin was used as starting material for the production of GelMA. The protein was dissolved in a CB buffer solution at pH 9. After the complete dissolution of the gelatin, MAA was added continuously. Both steps were performed under thorough stirring at 50 ${}^{\circ }C$. After the reaction was stopped, the purification of the GelMA took place using dialysis tubing in an ultrapure water reservoir at 40 ${}^{\circ }C$. A second approach for the dissolution and synthesis of GelMA was performed. For this purpose, gelatin was dissolved at RT in a CB buffer at pH 9 that contained 4 M urea. The dissolution of gelatin in the urea-containing buffer solely required stirring. The presence of urea in the buffer also allowed the efficient mixing of MAA during the synthesis, which was also performed at RT. In contrast, the purification set-up for this approach did require the heating of the TFF unit to 50 ${}^{\circ }C$. The DoF of GelMA produced at MAA-to-gelatin ratio of 100 μL g^−1^ was determined. GelMA synthesized in the CB buffer (50C100MA) exhibited a DoF of 0.926 ± 0.057 and did not significantly differ from GelMA produced in the urea containing CB buffer (RT100MA), which showed a DoF of 0.963 ± 0.027, as presented on Table 1 and Figure 3.

As MAA reacts with neutral free amine groups, a pH value higher than the isoelectric point of gelatin had to be maintained during the reaction. The typical isoelectric point of type A gelatin lies between pH 7 and pH 9. Therefore, the functionalization process was performed at pH 9 in a buffered solution, similar to the methods by Lee [17] and Shirahama [18]. These and previous studies report the processing of gelatin at temperatures above the gelation point of gelatin solutions [8,20,21]. A brief overview of the synthesis parameters found in the literature is schematically shown in Figure 1. Gelatin undergoes a sol–gel transition due to inter- and intramolecular interactions of the biopolymer as the protein backbone forms helical structures similar to those found in collagen [22,23]. In order to process gelatin at RT, urea was used in the reaction buffer. Urea causes the unfolding of proteins as the hydrophobic interaction between protein chains is disrupted [24,25,26]. This effect was used in order to ensure the sol state at RT, thus enabling the homogeneous mixing of MAA during the synthesis of GelMA. The purification of GelMA produced at RT required heating of the complete TFF set-up, GelMA, and water reservoirs, as well as the membrane. The decreasing concentration of urea during purification allows the re-folding of GelMA protein coils to helical structures, i.e., the transition from a solution to a gel [8]. Nevertheless, the purification time was reduced to about ten hours compared to the several days required by the dialysis process. It is noteworthy to mention that the purity of the samples should be analyzed using other methodologies in future studies, as in the presented study the purification was indirectly controlled by measuring the conductivity of the liquid used during purification, and no direct quantification of the purity of the sample was performed. In addition, there were no significant differences between the DoF of GelMA produced by both methods, and the values are in the same range as previous studies [17,18,20]. Additionally, Shirahama [18] also showed that the produced GelMA was not influenced by the reaction temperature in the range from 35 to 50 ${}^{\circ }C$. The DoF of GelMA produced at higher temperatures is comparable to the DoF of GelMA synthesized by the presented approach at RT; thus, this finding could indicate that the reaction kinetics is controlled by the molecular weight and molecular weight distribution of the used gelatin, rather than by the reaction temperature. Differences in molecular weight can arise not only from the species of the gelatin source but also from the bloom strength and even from batch-to-batch variations of the same gelatin product. The DoF of type B GelMA produced with bovine gelatin has been studied in the literature showing a higher value than that of type A GelMA produced with porcine gelatin [27]. This is due to the fact that the pH of the buffered solution remains above the IEP of bovine gelatin during the reaction. The presented method of reaction buffer containing urea should also be implemented in future studies for the comparison of the properties of GelMA from different species as well as varying bloom strength.

The effect of the MAA-to-gelatin ratio was also studied for the developed synthesis at RT and is shown in Figure 3. All synthesized batches are summarized in Table 1. The ratio was varied from 12.5 to 100 μL g^−1^, where the increasing ratio leads to higher DoF ranging from 0.044 ± 0.028 to 0.963 ± 0.027. Statistically significant differences between the DoF values were found for all data sets (p<0.05). The effect of the increasing ratio is in accordance with other studies [17,18,20,21]. Based on the reactant ratio, the resulting GelMA product can be controlled in a reproducible manner as required for the formulation of hydrogels.

### 3.2. Hydrogel Characterization

**Figure 4:**
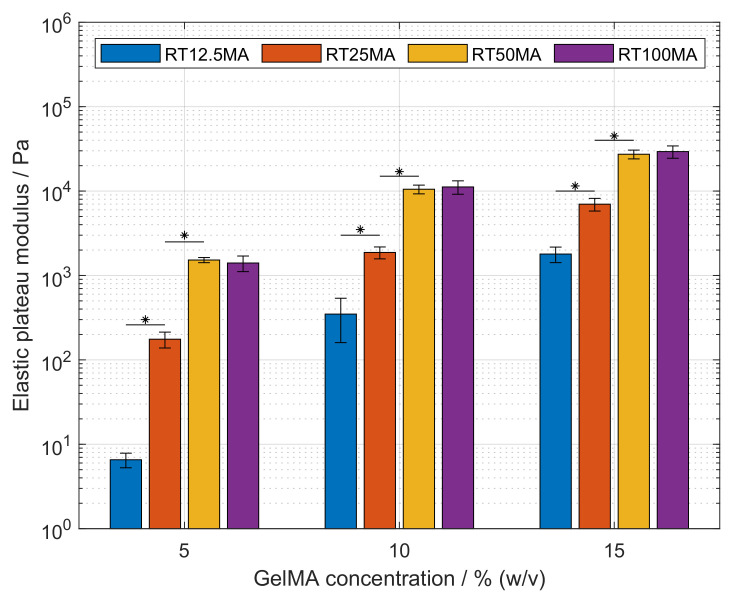
Elastic plateau modulus of gelatin methacryloyl (GelMA) hydrogels at various concentrations. The elasticity of hydrogels increased significantly with higher GelMA concentrations of the same synthesized sample (p<0.05). These differences are not shown for the purpose of clarity. Significant differences between the elastic modulus of samples at a constant GelMA concentration are denoted with an asterisk (p<0.05).

GelMA hydrogels can be covalently crosslinked to generate hydrogels with structural properties according to the required application. In cell culture, the physical properties of the matrix environment are known to influence cellular migration, proliferation, and differentiation [28,29,30]. Hydrogels were prepared as described previously and the resulting storage modulus was determined by oscillatory frequency sweeps on a rheometer. Holding the DoF constant, the elastic modulus showed a significant increase with higher polymer concentration (p<0.05), as shown in Figure 4. The moduli of RT100MA hydrogels at 5, 10 and 15% (*w*/*v*) were 1.4, 11.2 and 29.4 kPa, respectively. The same trend was observed with all used samples, i.e., RT50MA, RT25MA, and RT12.5MA. The increase of the elastic modulus corresponds to a higher crosslink density in the polymeric network [31]. The higher GelMA content leads to an increased availability of crosslinking sites as well as an increasing amount of physical entanglements. This behavior is comparable with other studies [27,32,33].

The elasticity of the hydrogels was influenced by the DoF, this effect is presented in Figure 4 as well. Hydrogels prepared from the samples RT50MA, RT25MA, and RT12.5MA with DoFs of 0.657, 0.176 and 0.044, respectively, show a significant increase of elasticity with increasing DoF at a constant GelMA concentration (p<0.05). At a concentration of 15% (*w*/*v*), the moduli increased from 1.8–29.4 kPa with increasing DoFs. The elasticity of hydrogels produced from RT100MA and RT50MA was in the same range and did not differ significantly at any tested GelMA concentration, even though the DoFs of both samples differed significantly. The DoF of GelMA RT100MA was 0.963, while the DoF of the RT50MA sample was 0.657.

The results observed from the samples RT50MA, RT25MA, and RT12.5MA are in accordance with other studies [27,32,33]. The increasing elasticity of the hydrogels at a constant polymer concentration is attributed to the higher crosslink density proceeding from the higher amount of methacrylamide and methacrylate residues. The missing difference between the samples prepared with RT50MA and RT100MA could arise from the crosslinking condition used in this study. The irradiation dose was set to 2850 mJ cm^−2^ and the concentration of the photo-initiator was set to 0.5% (*w*/*v*). The irradiance dose is higher than those used in similar studies by Van Den Bulcke [8], Nichol [32], and Pepelanova [20] which were set to 10, 6.9 and 1200 mJ cm^−2^, respectively. The concentration of the used photo-initiator was also higher than the concentration presented by Van Den Bulcke (0.006% (*w*/*v*)) [8], Schuurman (0.05% (*w*/*v*)) [12], and Lee (0.1% (*w*/*v*)) [27]. In free radical polymerization, the reaction rate is proportional to the irradiance and photo-initiator concentration, as studied by O’Connell [33]. The fast generation of free radicals, which initiates the chain polymerization, is opposed by the increasing elasticity of the matrix, i.e., the decreasing mobility of the polymeric network and the lower diffusivity of free radicals. Thus, reaching a limit of the created crosslinks.

**Figure 5:**
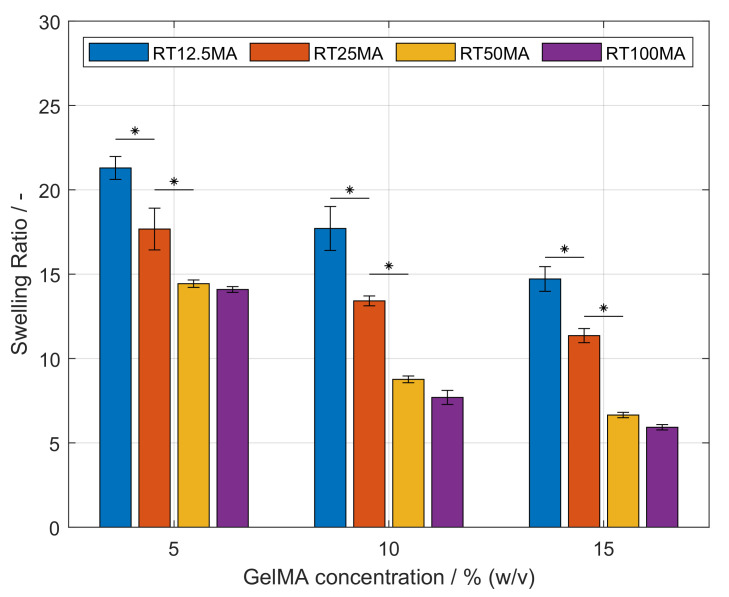
Equilibrium swelling ratio of gelatin methacryloyl (GelMA) hydrogels at various concentrations. The swelling behavior increased significantly with higher GelMA concentrations of the same synthesized sample (p<0.05). These differences are not shown for the purpose of clarity. Significant differences between the swelling ratio of samples at a constant GelMA concentration are denoted with an asterisk (p<0.05).

The swelling behavior of hydrogels reflects the ability of the polymeric network to bind and retain aqueous media. This property influences the diffusion of nutrients to the cells and metabolic by-products away from the cells [34]. As shown in Figure 5, it was observed that the swelling ratio of hydrogels was influenced by the used GelMA concentration as well as the DoF. Maintaining a constant DoF, an increasing amount of GelMA significantly reduced the swelling capacity of the network (p<0.05). Furthermore, a higher DoF led to lower swelling ratios at a constant concentration. This effect was significant between the samples RT50MA, RT25MA, and RT12.5MA (p<0.05). Similar to the observation during the characterization of the mechanical properties, the swelling ratios of hydrogels produced with GelMA RT50MA and RT100MA did not differ significantly. The effect of GelMA concentration and DoF on the swelling capacity of GelMA hydrogels have also been demonstrated by other studies [11,32,35,36] and are in agreement with the observations presented in this study. The driving mechanism of swelling is the osmotic pressure difference between the fluid within the network and the outer solution, which is opposed by the elasticity of the network preventing the dissolution due to its crosslinked structure [34,37]. Therefore, more elastic hydrogels produced with higher GelMA concentrations or higher DoFs have a lower swelling capability due to the higher crosslink density. As previously described, the similarity between the swelling ratio of the hydrogel samples RT50MA and RT100MA could be explained by the crosslinking conditions. It has to be kept in mind that the physical properties of a crosslinked GelMA hydrogel also depend on the properties of the used gelatin before functionalization such as the animal source, the bloom strength, and the crosslinking conditions [20,27,38]. By variation of the gelatin bloom strength, and, therefore, variation of the molecular weight of the biopolymer, the range of elasticity of the produced hydrogels could be expanded in further investigations. The GelMA hydrogels produced in this study proved to be suitable for mimicking the physical properties of tissue. Such properties can be adjusted in a controllable manner over the concentration of biopolymer and/or DoF in order to adjust the hydrogel to the specific cell type requirements.

### 3.3. Biocompatibility Assessment

GelMA hydrogels were used as carriers for NIH 3T3 fibroblasts in culture. The biocompatibility of synthesized GelMA as a hydrogel was determined via quantification of the cell viability after one, and after three days in cultivation. For this purpose, fluorescent staining and subsequent imaging of the samples was performed. The acquired images were imported into Matlab^®^ and evaluated using the image processing workflow, as described in Section 2.3.4. During processing, the detected signals were binarized separately. Single nuclei in contiguous regions were identified using a watershed segmentation tool. Nuclei of cells under the binary mask proceeding from the green channel were counted as live cells, whereas nuclei under the red mask were classified as dead cells. The image processing workflow enabled the objective and automated analysis of the gained frames. The developed tool is intended to reduce the time required for the analysis of images. Moreover, the automated identification of cells is also advantageous as it increases reproducibility and reduces observer-dependent errors [39,40,41]. The used image processing workflow allowed the analysis of relatively large data sets, which consisted of at least 40 frames with more than 1500 identified cells for each tested material. In contrast, similar studies in the field of tissue engineering and biofabrication are limited to a relatively low number of counted cells [27,42] and/or a low number of acquired images by microscopy [12,43].

**Figure 6:**
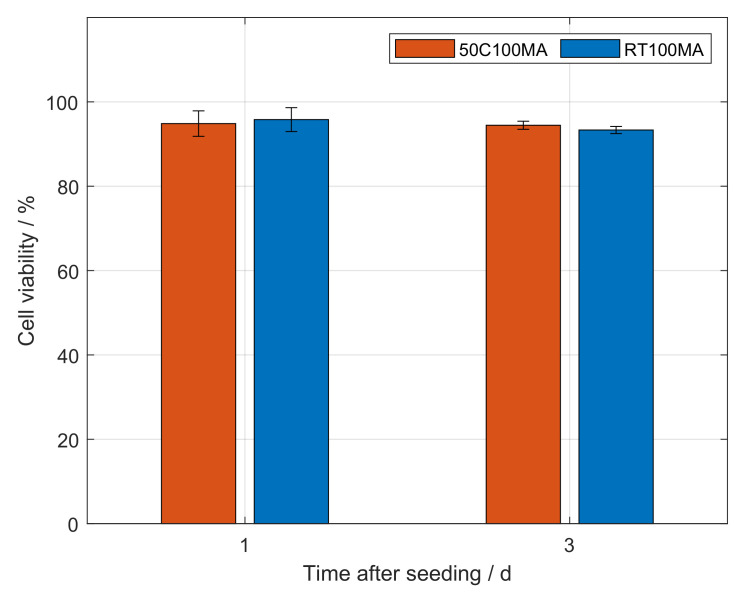
Cell viability of NIH 3T3 fibroblasts on gelatin methacryloyl (GelMA) coated well plates. GelMA produced similar to the method of Shirahama [18] at 50 ${}^{\circ }C$ (50C100MA) was used as a control in comparison with GelMA produced by the novel approach presented in this study, i.e., at room temperature (RT100MA). The viability of cells seeded onto both hydrogels samples did not differ significantly after one and three days.

Figure 6 shows the viability of cells seeded onto 10% (*w*/*v*) GelMA hydrogels. Cells growing on GelMA synthesized according to the conventional method at 50 ${}^{\circ }C$ (50C100MA) showed viabilities of 94.8% and 94.5% after one and three days, respectively. The viability of cells on GelMA produced at room temperature (RT100MA) was quantified to a value of 95.8% after one day of incubation, and a value of 93.3% after three days.

The viability of cells after one day did not significantly differ from the viability after three days for either hydrogel. Hence, no significant difference in viability was observed between cells growing on GelMA 50C100MA and on GelMA RT100MA. This approach of GelMA production at room temperature enabled the synthesis of a biocompatible material for hydrogel formulation. The high viability is in accordance with similar studies that use GelMA produced following conventional methods [27,32,38,42]. In the field of tissue engineering, the applications of hydrogels also include cell growth within the produced scaffold as well as cellular invasion in scaffolds. Such studies should be performed with GelMA produced according to the presented methodology.

## 4. Conclusions

In the field of tissue engineering, the use of GelMA-based hydrogels is well established due to the biocompatible nature of the biomaterial. The current manufacturing of products containing gelatin and gelatin derivatives requires heating, as gelatin solutions undergo a transition to a gel state at lower temperatures. This study presents a novel approach to functionalize gelatin to GelMA at room temperature. This process was possible due to the presence of urea in the synthesis buffer, as urea decreases the protein–protein interaction, thereby inhibiting the gelation of the solution and allowing efficient mixing of the reactants. GelMA with different degrees of functionalization was produced in a controllable manner by variation of the reactant ratio. By variation of the concentration of GelMA and its DoF, hydrogels were prepared with elastic properties in the range of 1.8 to 24 kPa, thus enabling the precise adaptation to specific cell type requirements. Moreover, the determination of cell viability was performed by imaging and a subsequent image processing workflow that allowed the time-saving analysis of several frames. GelMA produced at room temperature proved to be suitable for cell culture applications with the cell line NIH 3T3 and no difference was observed in comparison to GelMA produced by the conventional method at elevated temperatures, i.e., 50 ${}^{\circ }C$. The possibility to manufacture GelMA at room temperature shows several advantages. First, gelatin solutions are prone to the growth of microorganisms and would require cooling during longer transition periods between operations such as gelatin dissolution and GelMA synthesis. The presence of urea in the solution can be helpful during mentioned time intervals as urea is known to inhibit microbial growth. Second, the growing demand for biomaterials and related products such as bioinks requires suitable means to produce them efficiently in large quantities. Processing at room temperature, i.e., without heating, could facilitate the transition to large-scale production as undesired temperature profiles in reactors can lead to poor mixing and improper distribution of reactants, thus leading to non-reproducible processes. Thirdly, and of significant importance, the production of GelMA for clinical stages might face conditions imposed by regulatory agencies. These require detailed information on the range of operating conditions that will result in the production of materials meeting quality criteria. In the case of GelMA, the presented approach widens the operating range in terms of temperature in order to achieve the aimed degree of functionalization, and, therefore, the robustness of the synthesis process is enhanced.

## Figures and Tables

**Table 1:** Overview of the experimental set-up for the synthesis of gelatin methacryloyl (GelMA), and the sample nomenclature used throughout this manuscript. CB: carbonate-bicarbonate, DoF: degree of functionalization RT: room temperature.

Synthesis Buffer	MAA-to-Gelatin Ratio μL/g	Reaction Temperature	DoF -	Nomenclature
0.25 M CB	100	50 ${}^{\circ }C$	0.926	50C100MA
0.25 M CB, 4 M urea	100	RT	0.963	RT100MA
0.25 M CB, 4 M urea	50	RT	0.657	RT50MA
0.25 M CB, 4 M urea	25	RT	0.176	RT25MA
0.25 M CB, 4 M urea	12.5	RT	0.044	RT12.5MA

## Data Availability

The raw data supporting the conclusions of this article as well as the written codes for Matlab^®^ will be made available on request. Inquiries can be directed to the corresponding author.

## Abbreviations

The following abbreviations are used in this manuscript:

CAM	Calcein-AM
DoF	Degree of functionalization
ECM	Extracellular matrix
GelMA	Gelatin-methacryloyl
IEP	Isoelectric point
MAA	Methacrylic anhydride
PI	Propidium iodide
TE	Tissue engineering
TFF	Tangential flow filtration
TNBS	2,4,6-Trinitrobenzenesulfonic acid solution
